# Laparoscopic distal gastrectomy demonstrates acceptable outcomes regarding complications compared to open surgery for gastric cancer patients with pylorus outlet obstruction

**DOI:** 10.3389/fonc.2023.1169454

**Published:** 2023-04-27

**Authors:** Sen Wang, Yigang Zhang, Zetian Chen, Qingya Li, Fengyuan Li, Zheng Li, Hongda Liu, Zhe Xuan, Yiwen Xia, Penghui Xu, Lang Fang, Linjun Wang, Diancai Zhang, Hao Xu, Li Yang, Zekuan Xu

**Affiliations:** ^1^ Department of General Surgery, The First Affiliated Hospital of Nanjing Medical University, Nanjing, China; ^2^ Collaborative Innovation Center For Cancer Personalized Medicine, Nanjing Medical University, Nanjing, China; ^3^ The Institute of Gastric Cancer, Nanjing Medical University, Nanjing, China

**Keywords:** gastric cancer, pylorus outlet obstruction, laparoscopic distal gastrectomy (LDG), complications, post-operation hospital stay

## Abstract

**Background:**

For gastric cancer (GC) patients with pylorus outlet obstruction (POO), whether laparoscopic surgery has advantages over open surgery remains unclear. This study aims to investigate the differences between patients with and without POO in open and laparoscopic groups and to determine the differences between laparoscopic distal gastrectomy (LDG) and open distal gastrectomy (ODG) in GC patients with POO.

**Methods:**

A total of 241 GC patients with POO who underwent distal gastrectomy at the Department of Gastric Surgery of the First Affiliated Hospital of Nanjing Medical University between 2016 and 2021 were included in this study. A total of 1,121 non-POO patients who underwent laparoscopic surgery and 948 non-POO patients who underwent open surgery from 2016 to 2021 were also enrolled in the study. We compared complication rates and hospital stays between open and laparoscopic groups.

**Results:**

There was no significant difference for LDG between GC patients with and without POO regarding the overall complication rates (P = 0.063), the Grade III–V complication rate (P = 0.673), and the anastomotic complication rate (P = 0.497) from 2016 to 2021. The patients with POO had longer preoperative hospital stay (P = 0.001) and postoperative hospital stay (P=0.007) compared to patients without POO. No significant difference was observed for open patients between POO and non-POO patients regarding the overall complication rate (P = 0.357), grade III–V complication rate (P = 1.000), and anastomosis-related complication rate (P = 0.766). Compared with open surgery in GC patients with POO (n = 111), the total complication rate of the LDG group was 16.2%, which was significantly lower than that of the open group (26.1%, P = 0.041). No significant differences in the Grade III–V complication rate (P = 0.574) and anastomotic complication rate (P = 0.587) were observed between laparoscopic and open groups. Patients receiving laparoscopic surgery had shorter postoperative hospital stay than open surgery (P = 0.001). More resected lymph nodes (LNs) were also observed in the laparoscopic group (P = 0.0145).

**Conclusion:**

The comorbidity of GC with POO does not increase the complication rate after laparoscopic or open distal gastrectomy. In GC patients with POO, laparoscopic surgery shows advantages over open surgery with a lower overall complication rate, shorter postoperative hospital stay, and more harvested lymph nodes. Laparoscopic surgery is a safe, feasible, and effective treatment for GC with POO.

## Introduction

Gastric cancer (GC) is a common malignant tumor of the digestive system. Currently, GC, so far, ranks fifth in global incidence and fourth in global cancer–related mortality (1). As a country with a large population of GC, China has highest number of new GC cases and mortality globally per year (2). As GC progresses, patients with tumors located in the lower part of the stomach may develop pyloric outlet obstruction (POO), which worsens the primary disease and makes treatment more challenging. POO is caused by the mechanical blockage of gastric emptying, often resulting from advanced GC, and patients with POO usually have poor health conditions (3). Typical symptoms of POO include abdominal pain, nausea, and intractable vomiting, which severely affect the quality of life for patients and usually present no specific clinical manifestation compared with other benign obstructions (4). In addition, the edema of the gastric wall could be caused by the accumulation of food in stomach; thus, it is speculated that a high complication rate is associated with gastric surgery on POO patients due to poor nutrition and advanced tumor progress in these patients (5). While 15%–20% of GC patients may develop POO, the incidence could be higher in China due to the high prevalence of advanced GC (6, 7).

Current treatment for GC patients with pyloric outlet obstruction includes surgery, duodenal stent (8–11), and endoscopic ultrasonography–guided gastroenterostomy (12–14). However, considering the balance between the treatment of the primary malignancies and the remission of obstructive symptoms, surgery is still recommended as the primary treatment for GC with POO, which includes laparoscopic and traditional open surgery. In our center, a full preoperation treatment is administrated to patients with POO, including gastric lavage, nutrition support, and other managements to ensure that operable patients with POO undergo radical surgical intervention. Distal gastrectomy still remains the main method of clinical treatment for distal GC with POO (15). However, whether laparoscopic distal gastrectomy (LDG) and open distal gastrectomy (ODG) are associated with higher complication rates for patients with POO compared to patients without POO is still controversial. Kuroda et al. reported that POO would increase the postoperative complications, while other reports suggested that POO was not associated with higher postoperative complication rates (5, 16, 17). Moreover, whether LDG has advantages over ODG in GC patients with POO is still unclear and no consensus was reached on this issue.

This study aims to evaluate and compare differences between patients with and without POO in open and laparoscopic groups and to determine the differences between LDG and ODG regarding complication rates and the length of hospital stay in the treatment of GC patients with POO.

## Methods

### Patients and treatments

We retrospectively reviewed all cases of GC with POO being treated in the Department of Gastric Surgery of the First Affiliated Hospital of Nanjing Medical University between January 2016 and December 2021. The patient selection criteria were as follows. Inclusion criteria were patients aged 18–80 years who underwent open or laparoscopic distal gastrectomy with D2 lymph node dissection; gastric adenocarcinoma identified based on complete and clear pathologic information from the American Joint Committee on Cancer (AJCC) pathologic Tumor Node Metastasis (TNM) staging system; preoperative endoscopic diagnosis of POO; and positive symptoms of vomiting or difficulty in food intake. The criteria of POO include positive results of endoscopic examination or CT scan. Surgeons with over 50 cases of laparoscopic distal gastrectomy were enrolled in this study to reduce the bias of the surgical experience of different surgeons. Exclusion criteria were GC with synchronous malignancy or major organ resections. Palliative operations and patients with neoadjuvant chemotherapy were also ruled out. During this period, all GC patients were treated in accordance with Japanese guidelines before and after surgery (18). For preoperative treatment, POO patients were fasted and administrated with a gastric tube for enteral nutrition support. All anastomoses were selected based on the clinical stage of tumor, the location of the tumor, and the experience and preference of different surgeons.

### Complications

The complication categories include duodenal stump leakage, anastomotic leakage, anastomotic bleeding, intraabdominal bleeding, intestinal obstruction, abdominal infection, seroperitoneum, wound infection, lymphatic fistula, anemia, mobility disorder, cardiac complications, pulmonary complications, urinary and renal complications, hepatobiliary complications, other gastrointestinal complications, and thrombosis. Complications after surgery within 30 days were assessed and prospectively collected by the residents and statisticians. The complication’s severity was classified according to the Clavien–Dindo classification system. All complications were treated accordingly.

### Statistical analysis

Mann–Whitney and t-test analyses are used throughout the study. Continuous variables are described as the mean ± SD or median (Q1 and Q3), and categorized variables are summarized by frequency (n) and proportion (%). The chi-square test was used for rate or proportion comparison. Statistical analyses were performed using IBM SPSS version 25.0. The univariate and multivariate analyses of variance was conducted by R software. Written consent was obtained from all patients in the First Affiliated Hospital of Nanjing Medical University (NMUH), and it was approved by the hospital’s ethics committee.

## Results

### Pyloric outlet obstruction would not increase complication rates after laparoscopic surgery

A total of 130 GC patients with POO who underwent LDG and 111 GC patients with POO who underwent ODG met the criteria for the inclusion criteria of this study. The basic characteristics of enrolled patients are listed in **Table 1**.

**Table 1:** Basic characteristics of gastric cancer (GC) patients with pyloric outlet obstruction (POO).

Variable	Total (n = 241)	Laparoscope (n = 130)	Open (n = 111)	P-value
Age	64.44 ± 11.01	65.27 ± 11.18	63.46 ± 10.78	0.204
Gender				0.487
Male	165 (68.5%)	86 (66.2%)	79 (71.2%)	
Female	76 (31.5%)	44 (33.8%)	32 (28.8%)	
Anastomosis				**<0.001**
B-I	10 (4.1%)	3 (2.3%)	7 (6.3%)	
B-II	114 (47.3%)	91 (70.0%)	23 (20.7%)	
R-Y	90 (37.3%)	11 (8.5%)	79 (71.2%)	
Uncut	27 (11.3%)	25 (19.2%)	2 (1.8%)	
T stage				**<0.001**
1a	9 (3.7%)	7 (5.4%)	2 (1.8%)	
1b	8 (3.3%)	5 (3.8%)	3 (2.7%)	
2	12 (5.0%)	7 (5.4%)	5 (4.5%)	
3	98 (40.7%)	71 (54.6%)	27 (24.3%)	
4a	110 (45.6%)	38 (29.2%)	72 (64.9%)	
4b	4 (1.7%)	2 (1.5%)	2 (1.8%)	
N stage				0.511
0	61 (25.3%)	35 (26.9%)	26 (23.4%)	
1	26 (10.8%)	17 (13.1%)	9 (8.1%)	
2	43 (17.8%)	23 (17.7%)	20 (18.0%)	
3a	62 (25.7%)	33 (25.4%)	29 (26.1%)	
3b	49 (20.4%)	22 (16.9%)	27 (24.4%)	

Bold values indicate that the data is statistically significant.

In order to detect whether POO would be positively associated with higher overall complications, grade III–V complications, and anastomosis-related complications after LDG, we first compared the overall complication rates between GC patients who underwent LDG with and without POO from 2016 to 2021 in our center. The results demonstrated that even very close, the overall complication rate of LDG in POO patients was 16.2% (21/130), which was not significantly higher than that of cases without POO (10.7%, 120/1,121, P = 0.063, **Table 2**). Regarding grade III–V complication rates, GC patients with POO were not dramatically different from those without POO [3.8% (5/130) vs. 2.8% (31/1,121), P = 0.673, **Table 2**]. We next investigated whether LDG would be associated with anastomosis-related complications. Based on our experience and publications, we defined anastomosis leakage, anastomosis bleeding, and duodenal stump burst as anastomosis-related complications. We found that there was no significant difference between the POO group and the without POO group [3.1% (4/130) vs. 1.8%, 20/1,121, P = 0.497, **Table 2**]. The above findings indicated that LDG is a safe procedure to treat GC patients with POO, which would not increase the overall complication rate, grade III–V complication rate and anastomosis-related complication rate in our center compared to patients without POO, which might not be consistent with common expectations.

**Table 2:** The information of complication rates, hospital stays, and resected lymph nodes (LNs) between POO and non-POO patients of laparoscopic cases from NMUH.

	Anastomosis	Laparoscope POO (n = 130)	Laparoscope without POO (n = 1,121)	P- value
Overall complication rates	B-I	0/3 (0%)	2/12 (16.7%)	
	B-II	15/91 (16.5%)	76/686 (11.1%)	
	R-Y	3/11 (27.3%)	27/187 (14.4%)	
	Uncut	3/25 (12.0%)	15/236 (6.4%)	
	Overall	21/130 (16.2%)	120/1,121 (10.7%)	0.063
Grade III–V complication rates	B-I	0/3 (0%)	2/12 (16.7%)	
	B-II	3/91 (3.3%)	19/686 (2.8%)	
	R-Y	0/11 (0%)	6/187 (3.2%)	
	Uncut	2/25 (8.0%)	4/236 (1.7%)	
	Overall	5/130 (3.8%)	31/1,121 (2.8%)	0.673
Anastomosis-related Complication rates	B-I	0/3 (0%)	1/12 (8.3%)	
	B-II	2/91 (2.2%)	9/686 (1.3%)	
	R-Y	1/11 (9.1%)	8/187 (4.3%)	
	Uncut	1/25 (4.0%)	2/236 (0.8%)	
	Overall	4/130 (3.1%)	20/1,121 (1.8%)	0.497
Preoperation hospital stay	Overall	8 (7, 9)	5 (3, 6)	**0.001**
Postoperation hospital stay	Overall	8 (7, 10)	7 (7, 8)	**0.007**
Resected LNs	Overall	42.2 ± 11.9	42.3 ± 11.1	0.883

Bold values indicate that the data is statistically significant.

### Pyloric outlet obstruction was associated with longer postoperative stay and not associated with higher lymph nodes in laparoscopic distal gastrectomy

Within expectation, the length of the preoperative hospital stay of GC patients with POO was significantly longer than that of those without POO [8 (7, 9) days vs. 5 (3, 6) days, P = 0.001, **Table 2**), which was probably due to the preoperative management of POO such as gastric lavage. We then noticed shorter postoperative hospital stays for patients without POO than patients with POO after laparoscopic treatment [8 (7, 10) days vs. 7 (7, 8) days, P = 0.001, **Table 2**]. This result suggested that POO would still extend the length of postoperative hospital time. Then, we compared the number of lymph node dissection between the above two groups and no significant difference was discovered (42.2 ± 11.9 vs. 42.3 ± 11. 1, P = 0.883, **Table 2**), which demonstrated that POO did not decrease the harvested LNs during the LDG procedure and confirmed again that laparoscopic surgery would not only be safe but also quality-guaranteed for GC patients with POO.

### Pyloric outlet obstruction was not associated with higher complication rates and resected lymph nodes in open distal gastrectomy

Next, we did a similar analysis between GC patients who underwent ODG with and without POO from 2016 to 2021 in our center (**Table 3**). The results showed that there was no significant difference in the overall complication rate between POO patients (26.1%, 29/111) and non-POO patients (22.3%, 211/948) who underwent open surgery (P = 0.357, **Table 3**). No significant difference was observed between POO and non-POO patients, on grade III–V complication rates (2/111, 1.8% vs. 21/948, 2.2%, P = 1.000) and anastomosis-related complications (3/111, 2.7% vs. 17/948, 1.8%, P = 0.766) either. These results indicated that POO was not associated with higher overall complication rates, grade III–V complication rates, and anastomosis-related complication rates in open surgery.

**Table 3:** The information of complication rates, hospital stays and resected LNs between POO and non-POO patients of open cases from NMUH.

	Anastomosis	Open with POO (n = 130)	Open without POO (n = 948)	P-value
Overall complication rates	B-I	1/7 (14.3%)	2/8 (25.0%)	
	B-II	6/23 (26.1%)	29/118 (25.6%)	
	R-Y	22/79 (27.8%)	176/799 (22.0%)	
	Uncut	0/2 (0%)	4/23 (17.4%)	
	Overall	29/111 (26.1%)	211/948 (22.3%)	0.357
Grade III–V complication rates	B-I	0/7 (0%)	0/8 (0%)	
	B-II	0/23 (0%)	5/118 (4.2%)	
	R-Y	2/79 (2.5%)	16/799 (2.0%)	
	Uncut	0/2 (0%)	0/23 (0%)	
	Overall	2/111 (1.8%)	21/948 (2.2%)	1.000
Anastomosis-related complication rates	B-I	0/7 (0%)	0/8 (0%)	
	B-II	0/23 (0%)	3/118 (2.6%)	
	R-Y	3/79 (3.8%)	14/799 (1.7%)	
	Uncut	0/2 (0%)	0/23 (0%)	
	Overall	3/111 (2.7%)	17/948 (1.8%)	0.766
Preoperation hospital stay	Overall	8 (7, 9)	5 (4, 6)	0.001^*^
Postoperation hospital stay	Overall	11 (9, 13)	10 (9, 12)	0.222
Resected LNs	Overall	42.2 ± 11.9	39.8 ± 11.7	0.228

Bold values indicate that the data is statistically significant.

### Pyloric outlet obstruction was not associated with longer postop stay after open distal gastrectomy

Similarly, the preop hospital stay of patients without POO was significantly shorter than POO patients [5 (4, 6) vs. 8 (7, 9), P = 0.001, **Table 3**], which was within the expectation, while the postop stay did not differ from each other [10 (9, 12) vs. 11 (9, 13), P = 0.222). Regarding harvested LNs, no difference was observed between POO (42.2 ± 11.9) and non-POO patients (39.8 ± 11.7, P = 0.228). These results also suggested that POO might not influence the quality of surgery in ODG, which was similar to the laparoscopic group analysis.

### Laparoscopic surgery decreased the overall complication rate in gastric cancer patients with pyloric outlet obstruction compared to open distal gastrectomy

We then reviewed 111 GC cases with POO who underwent ODG in our center from 2016 to 2021 for analysis. The total complication rate of open surgery was 26.1% (29/111), which was significantly higher than that of laparoscopic surgery (16.2%, 21/130, P = 0.041, **Table 4**). We also performed univariate and multivariate analyses of variance to investigate the influence of different factors. Both univariate and multivariate analyses indicated that the laparoscopic method showed significant difference from ODG in the overall complication rate (**Supplementary Table 1**), which was consistent with previous results mentioned above. According to the analysis, the T stage and N stage of tumors had no impact on the overall complication rate, indicating that outcomes might only result from the selection of open or laparoscopy rather than different stages of tumors (**Supplementary Table 1**).

**Table 4:** The overall information of complication rates and hospital stays of all enrolled POO cases between open and laparoscopic groups from NMUH.

	Anastomosis	Laparoscope (n = 130)	Open (n = 111)	P-value
Overall complication rates	B-I	0/3 (0%)	1/7 (14.3%)	0.700
	B-II	15/91 (16.5%)	6/23 (26.1%)	0.219
	R-Y	3/11 (27.3%)	22/79 (27.8%)	0.639
	Uncut	3/25 (12.0%)	0/2 (0%)	0.786
	Overall	21/130 (16.2%)	29/111 (26.1%)	**0.041**
Grade III–V complication rates	B-I	0/3 (0%)	0/7 (0%)	NA
	B-II	3/91 (3.3%)	0/23 (0%)	0.798
	R-Y	0/11 (0%)	2/79 (2.5%)	0.769
	Uncut	2/25 (8.0%)	0/2 (0%)	0.855
	Overall	5/130 (3.8%)	2/111 (1.8%)	0.574
Anastomosis-related complication rates	B-I	0/3 (0%)	0/7 (0%)	NA
	B-II	2/91 (2.2%)	0/23 (0%)	0.636
	R-Y	1/11 (9.1%)	3/79 (3.8%)	0.412
	Uncut	1/25 (4.0%)	0/2 (0%)	0.926
	Overall	4/130 (3.1%)	3/111 (2.7%)	0.587
Preoperation Hospital stay	Overall	8 (7, 9)	8 (7, 9)	**0.130**
Postoperation hospital stay	Overall	8 (7, 10)	11 (9, 13)	**<0.001**

Bold values indicate that the data is statistically significant.

Next, we analyzed grade III–V complications rates between open and laparoscopic groups. We found that laparoscopic surgery did not differ from open surgery regarding grade III–V complications (5/130, 3.8% vs. 2/111, 1.8%, P = 0.574, **Table 4**), indicating that no severe postoperative complications were associated with laparoscopic or open procedure. The univariate and multivariate analyses of variance showed that no significant difference was observed associated with grade III–V complications (**Supplementary Table 2**). Although in the univariate analysis, the T stage might have an impact, the multivariate analysis confirmed that the grade III–V complication rate was not associated with the T stage. Moreover, no significant difference regarding the anastomotic complication rates (4/130, 3.1% vs. 3/111, 2.7%, P = 0.587, **Table 4**) were observed between laparoscopic and open groups. The univariate and multivariate analyses of variance again conformed that T and N stages would not affect anastomosis-related complications (**Supplementary Table 3**). These results indicated that the laparoscopic procedure might demonstrate better outcomes in overall complications compared to open surgery.

### Laparoscopy decreased the postoperative hospital stay in gastric cancer patients with pyloric outlet obstruction compared to open surgery

To detect whether the surgery method is associated with the length of hospital stay, we then analyzed the length of preoperative and postoperative hospital stays between laparoscopic and open surgery. The preoperative hospital stay of POO patients in the laparoscopic group was not significantly different from that in the open group [8 (7, 9) days vs. 8 (7, 9) days, P = 0.130, **Table 4**). For postoperative stay, patients who received laparoscopic surgery demonstrated significantly shorter postoperative stay than those with open surgery [8 (7, 10) days vs. 11 (9, 13) days, P = 0.0001, **Table 3**), indicating a better recovery of laparoscopic surgery.

Finally, we compared the number of lymph node dissections and operation time between the above two groups. More resected LNs were observed in the laparoscopic group compared to open surgery for GC patients with POO (42.2 ± 11.9 vs. 138.4 ± 12.1, P = 0.0145, **Table 5**). To compare the operation time between laparoscopic and open groups with POO, we discovered that laparoscopic surgery is significantly longer than open (185.9 ± 41.4 vs. 165.9 ± 52.9, P < 0.0001, **Table 5**), which was within our expectations.

**Table 5:** The overall information of resected LNs of all enrolled POO cases from NMUH.

		Laparoscope (n = 130)	Open (n = 111)	P-value
Resected LNs	Anastomosis			
	B-I	32.0 ± 17.4	21.1 ± 7.7	0.189
	B-II	43.1 ± 11.2	40.9 ± 10.6	0.393
	R-Y	44.2 ± 18.2	39.1 ± 11.9	0.218
	Uncut	39.1 ± 10.0	41.5 ± 3.5	0.745
	N-stage			
	0	38.2 ± 11.9	38.4 ± 12.1	0.960
	1	46.4 ± 16.8	35.4 ± 13.5	0.106
	2	42.0 ± 10.1	34.2 ± 16.6	0.066
	3a	43.2 ± 11.5	40.0 ± 10.1	0.239
	3b	43.7 ± 8.6	40.7 ± 9.5	0.248
	Overall	42.2 ± 11.9	38.4 ± 12.1	**0.0145**
Operation Time	Overall	185.9 ± 41.4	165.9 ± 52.9	**<0.001**

Bold values indicate that the data is statistically significant.

## Discussion

POO is comparatively common in GC patients, and the trends of GC with POO have continued to increase over the last decade. At present, it is generally believed that the occurrence of POO is closely related to cancer ulcer bleeding and edema, gastric wall contracture, and antrum tumor enlargement (19). In China, GC patients usually develop POO with growing symptoms such as vomiting, dehydration, and electrolyte imbalance and, when they come to hospital, these patients usually suffer from severe malnutrition. Long-term fasting will further worsen the condition (20). Surgery is not only a treatment but also an important stressor. Surgical trauma could activate the mononuclear-macrophage system, increase the number of neutrophils and white blood cells in the circulation and operative region, and release a large number of inflammatory mediators, which further affects the surgical effect and patient prognosis (21, 22). Traditional open surgery inflicts patients with a longer incision and larger surgical trauma. The bleeding can affect the vision of the operative field and cause unnecessary damage to the surrounding normal organs and tissues (23–25). Moreover, large surgical trauma leads to slow postoperative recovery, which affects the follow-up treatment. In recent years, many clinical trials (like JCOG1401, CLASS02, and KLASS03) reported that laparoscopic techniques with less surgical trauma have been widely accepted as a standard treatment for GC patients (26–28). The application rate of laparoscopic surgery in GC patients with pyloric obstruction has increased; however, the safety and feasibility need to be further verified in POO patients. This study shows that laparoscopic surgery is effective and safe in the treatment of GC with POO. We believe that the results of the present study offer significant insights into surgeons’ clinical treatment options.

We compared the complication rates and length of hospital stay between GC patients with and without POO who underwent LDG/ODG in our center. We found that the comorbidity of GC with POO was not associated with a higher overall complication rate, grade III–V, and anastomosis-related complication rates of LDG compared to normal GC patients without POO, indicating that POO might not affect complication rates in both LDG or ODG, which was not quite consistent with our previous expectations. Longer preoperative hospital stay was observed in the POO group, probably because patients with POO require more adequate preoperative preparations, such as gastrointestinal decompression and nutritional support. Preoperation nutritional support has become the routine step for GC patients with POO in our center, and we believe that it is very important for better recovery and fewer complications. We also noticed that the length of postop time in the LDG group was longer in the POO group than patients without POO. We assumed that although no significant difference was observed regarding the overall complication rate, it was very close to demonstrate a significant difference and that might affect the postoperative time in the LDG group. However, the difference of median time was only 1 day in the LDG group and it may also be biased by different discharging policies by different surgeons; thus, this might not be very clinically different. Generally, our results indicated that POO would not increase complication rates in both laparoscopic and open methods.

We then need to further discover whether laparoscopic surgery has advantages over open surgery regarding POO patients. We compared the complication rates and hospital stays between GC patients with POO who underwent laparoscopic or open surgery in our center. We reported that the total complication rate of the laparoscopic group was significantly lower than that of the open group. As the LOC-A Study demonstrated (25), the incidence of incision infection was lower in the laparoscopic group compared to open. Other studies have also already proven that the incidence of complications such as pancreatic fistula, the dumping syndrome, and postoperative ileus is higher in patients who underwent open surgery; thus, these results may partially explain why the overall complication rate of LDG was also lower than ODG in our study (29, 30).

Meanwhile, shorter postoperative hospital stay was observed in the laparoscopic surgery group. The lower overall complication might play a role in achieving a shorter postop stay in the laparoscopic group compared to open, as the difference of the median day between groups was not quite small and might be clinically different. We assumed that a smaller incision and less invasiveness might help faster recovery for patients with POO, for we confirmed that T and N stages would not affect the results. Thus, we might safely suggest that after full preoperation preparation, opting for an open approach or laparoscopy may lead to differences in the complications and lengths of postoperative stay.

Regarding the harvested lymph nodes, a statistical difference was observed between open and laparoscopic groups, suggesting a potential advantage of laparoscopy in harvested LNs due to the magnified surgical view and more precise operation. As a result, total operation time was consumed more in laparoscopic distal gastrectomy rather than open surgery, which was not beyond our expectations. Taken together, the aforementioned findings give us the confidence to believe that laparoscopic surgery is a safer, feasible, and effective therapeutic option for GC patients with POO over traditional open surgery.

As we mentioned above, current treatments for GC patients with POO not only include surgery; a duodenal stent with neoadjuvant chemotherapy is also one of the options for patients with advanced- stage GC. This method could also possibly help solve the difficulty in food intake with a comparatively easier procedure to place the stent and expect the downstaging of the tumor for possible future surgery. However, traditional duodenal stents could be comparatively easy to displace or migrate and result in the failure of food intake for patients; thus, in our center for GC patients with POO, surgery is usually the main treatment for operable patients. In our center, the laparoscopic approach usually comes to our first consideration for its better surgical view. Based on our experience in dissecting the No.6 LN area, dissecting right gastroepiploic vessels and infrapyloric vessels is one of the most important steps. Extreme caution should be taken to dissect these vessels, and it could be successfully dissected through precise anatomic layers. Thus, it is also critical to find correct anatomic layers of the No.6 LN area. The selection of the cartridge of a linear stapler is also important in treating POO patients. The cartridge (gold) of a closed stapler height 1.8 mm is usually used for transecting distal stomach for POO gastric cancer patients, while the cartridge (blue) of a closed stapler height 1.5 mm is applied for dissecting the duodenum. We also need to adjust the cartridge according to the extent of the edema of the pylorus. If the tumor is comparatively high, we routinely perform a ‘three-step’ purse suture on the duodenal stump to prevent leakage. If the tumor is located very low and edema is not relieved by preoperative management, the purse suture would be difficult to make; thus, we will reinforce the duodenal stump with a continuous suture using 4-0 suture material. All these procedures would help reduce the complication rates for POO patients, especially duodenum leakage. Certainly, LDG is still a technically challenging technique that requires a long learning curve, and only a well-trained laparoscopic surgeon can make the most of laparoscopic surgery. Thus, although our study provides insightful evidence for the application of laparoscopic surgery in complex GC patients like ones combined with POO, it also relies on experiences and preferences of different surgeons adopting a laparoscopic or open approach.

As we mentioned, we cannot ignore that the present study has several limitations. This study includes 241 GC cases with POO for a comparison between open and laparoscopic surgery, which is not a very big number, and since it is a retrospective study, it is inevitable that some cases would be missing. A large-scale prospective study with more enrolled cases might be needed. Also, with the development of staplers, suture materials, perioperation managements, and so on, these factors might also make a difference to our results. Moreover, the preference of the laparoscopic method by some certain surgeons might also bias the results, which is inevitable in the retrospective study.

## Conclusion

The comorbidity of POO with GC would not increase the complication rates after both LDG or ODG. In GC patients with POO, laparoscopic surgery shows advantages over open surgery with a lower overall complication rate, shorter postoperative hospital stay, and more harvested lymph nodes. Therefore, we believe that laparoscopic surgery is a safe, feasible, and effective treatment for GC with POO.

## Data availability statement

The raw data supporting the conclusions of this article will be made available by the authors, without undue reservation.

## Ethics statement

The studies involving human participants were reviewed and approved by the ethics committee of the First Affiliated Hospital of Nanjing Medical University. The patients/participants provided their written informed consent to participate in this study.

## Author contributions

SW designed the project and conducted the analysis and finished the manuscript writing. YZ and ZC helped in data analysis and manuscript writing. QL and FL collected data and helped in analysis. ZL, HL, YX, ZX, PX, and LF gave scientific suggestions to the project. LW, DZ, HX, and LY gave clinical guidance and professional suggestions. ZKX supervised and guided the project. All authors contributed to the article and approved the submitted version.

## References

[1] Sung H Ferlay J Siegel RL Laversanne M Soerjomataram I Jemal A. Global cancer statistics 2020: GLOBOCAN estimates of incidence and mortality worldwide for 36 cancers in 185 countries. CA: Cancer J Clin (2021) 71(3):209–49. doi: 10.3322/caac.21660 33538338

[2] Chen W Zheng R Baade PD Zhang S Zeng H Bray F. Cancer statistics in China, 2015. CA Cancer J Clin (2016) 66(2):115–32. doi: 10.3322/caac.21338 26808342

[3] Cheung SLH Teoh AYB. Optimal management of gastric outlet obstruction in unresectable malignancies. Gut liver (2022) 16(2):190–7. doi: 10.5009/gnl210010 PMC8924806 34039779

[4] Saeed SM Bilal S Siddique MZ Saqib M Shahid S Ghumman AN. Pyloric stent insertion in malignant gastric outlet obstruction: moving beyond palliation. Ther Adv gastrointest endosc (2021) 14:26317745211047012. doi: 10.1177/26317745211047012 34595475 PMC8477674

[5] Jiao X Wang Y Qu X Qu J Wang X. Effects of preoperative pyloric stenosis on outcomes and nutritional status in 73 patients following curative gastrectomy for gastric cancer: A retrospective study from a single center. Med Sci Monit (2021) 27:e930974. doi: 10.12659/MSM.930974 34315845 PMC8325391

[6] Papanikolaou IS Siersema PD. Gastric outlet obstruction: Current status and future directions. Gut Liver (2022) 16(5):667–75. doi: 10.5009/gnl210327 PMC9474481 35314520

[7] Lopera JE Brazzini A Gonzales A Castaneda-Zuniga WR. Gastroduodenal stent placement: current status. Radiographics (2004) 24(6):1561–73. doi: 10.1148/rg.246045033 15537965

[8] Boškoski I Tringali A Familiari P Mutignani M Costamagna G. Self-expandable metallic stents for malignant gastric outlet obstruction. Adv Ther (2010) 27(10):691–703. doi: 10.1007/s12325-010-0061-2 20737260

[9] Hirdes MM Vleggaar FP de Beule M Siersema PD. *In vitro* evaluation of the radial and axial force of self-expanding esophageal stents. Endoscopy (2013) 45(12):997–1005. doi: 10.1055/s-0033-1344985 24288220

[10] Hamada T Hakuta R Takahara N Sasaki T Nakai Y Isayama H. Covered versus uncovered metal stents for malignant gastric outlet obstruction: Systematic review and meta-analysis. Digest endosc (2017) 29(3):259–71. doi: 10.1111/den.12786 27997723

[11] Park JH Yoon H Kwak YJ Wang C Alzahrani KM Wang S. Feasibility and safety of inserting transient biodegradable stents in the pylorus during pylorus-preserving gastrectomy for gastric cancer: a preliminary study in a porcine for proof of concept. Gastric Cancer (2023) 26(1):155–66. doi: 10.1007/s10120-022-01350-5 36417001

[12] McCarty TR Garg R Thompson CC Rustagi T. Efficacy and safety of EUS-guided gastroenterostomy for benign and malignant gastric outlet obstruction: a systematic review and meta-analysis. Endosc Int Open (2019) 7(11):E1474–e82. doi: 10.1055/a-0996-8178 PMC6811354 31673620

[13] Wang G Liu X Wang S Ge N Guo J Sun S. Endoscopic ultrasound-guided gastroenterostomy: A promising alternative to surgery. J Trans Internal Med (2019) 7(3):93–9. doi: 10.2478/jtim-2019-0021 PMC6795053 31637179

[14] Irani S Itoi T Baron TH Khashab M. EUS-guided gastroenterostomy: techniques from East to West. VideoGIE (2020) 5(2):48–50. doi: 10.1016/j.vgie.2019.10.007 32051906 PMC7004893

[15] Ojima T Nakamori M Nakamura M Katsuda M Hayata K Yamaue H. Laparoscopic gastrojejunostomy for patients with unresectable gastric cancer with gastric outlet obstruction. J gastrointest Surg (2017) 21(8):1220–5. doi: 10.1007/s11605-017-3387-0 28224464

[16] Kuroda D Sawayama H Kurashige J Iwatsuki M Eto T Tokunaga R. Controlling nutritional status (CONUT) score is a prognostic marker for gastric cancer patients after curative resection. Gastric Cancer (2018) 21(2):204–12. doi: 10.1007/s10120-017-0744-3 28656485

[17] Park SH Mok YJ Kim JH Park SS Kim SJ Kim CS. Clinical significance of gastric outlet obstruction on the oncologic and surgical outcomes of radical surgery for carcinoma of the distal stomach. J Surg Oncol (2009) 100(3):215–21. doi: 10.1002/jso.21256 19235783

[18] Japanese Gastric Cancer A. Japanese Gastric cancer treatment guidelines 2021 (6th edition). Gastric Cancer (2023) 26(1):1–25. doi: 10.1007/s10120-022-01331-8 36342574 PMC9813208

[19] Miyazaki Y Takiguchi S Takahashi T Kurokawa Y Makino T Yamasaki M. Treatment of gastric outlet obstruction that results from unresectable gastric cancer: Current evidence. World J gastrointest endosc (2016) 8(3):165–72. doi: 10.4253/wjge.v8.i3.165 PMC4734975 26862366

[20] Troncone E Fugazza A Cappello A Del Vecchio Blanco G Monteleone G Repici A. Malignant gastric outlet obstruction: Which is the best therapeutic option? World J Gastroenterol (2020) 26(16):1847–60. doi: 10.3748/wjg.v26.i16.1847 PMC7201143 32390697

[21] Miller GD Nicklas BJ Fernandez A. Serial changes in inflammatory biomarkers after roux-en-Y gastric bypass surgery. Surg Obes related Dis (2011) 7(5):618–24. doi: 10.1016/j.soard.2011.03.006 PMC4811362 21546319

[22] Kristensen MD Lund MT Hansen M Poulsen SS Ploug T Dela F. Macrophage area content and phenotype in hepatic and adipose tissue in patients with obesity undergoing roux-en-Y gastric bypass. Obes (Silver Spring Md) (2017) 25(11):1921–31. doi: 10.1002/oby.21964 28921894

[23] Zeng YK Yang ZL Peng JS Lin HS Cai L. Laparoscopy-assisted versus open distal gastrectomy for early gastric cancer: evidence from randomized and nonrandomized clinical trials. Ann surg (2012) 256(1):39–52. doi: 10.1097/SLA.0b013e3182583e2e 22664559

[24] Chang KK Park do J Yoon SS. Laparoscopic versus open surgery for gastric adenocarcinoma: Innovation continues to challenge tradition. Ann surg (2016) 264(2):223–5. doi: 10.1097/SLA.0000000000001786 27163952

[25] Kinoshita T Uyama I Terashima M Noshiro H Nagai E Obama K. Long-term outcomes of laparoscopic versus open surgery for clinical stage II/III gastric cancer: A multicenter cohort study in Japan (LOC-a study). Ann surg (2019) 269(5):887–94. doi: 10.1097/SLA.0000000000002768 29697447

[26] Kataoka K Katai H Mizusawa J Katayama H Nakamura K Morita S. Non-randomized confirmatory trial of laparoscopy-assisted total gastrectomy and proximal gastrectomy with nodal dissection for clinical stage I gastric cancer: Japan clinical oncology group study JCOG1401. J gastric cancer (2016) 16(2):93–7. doi: 10.5230/jgc.2016.16.2.93 PMC4944008 27433394

[27] Liu F Huang C Xu Z Su X Zhao G Ye J. Morbidity and mortality of laparoscopic vs open total gastrectomy for clinical stage I gastric cancer: The CLASS02 multicenter randomized clinical trial. JAMA Oncol (2020) 6(10):1590–7. doi: 10.1001/jamaoncol.2020.3152 PMC7441466 32815991

[28] Yang HK Hyung WJ Han SU Lee YJ Park JM Cho GS. Comparison of surgical outcomes among different methods of esophagojejunostomy in laparoscopic total gastrectomy for clinical stage I proximal gastric cancer: results of a single-arm multicenter phase II clinical trial in Korea, KLASS 03. Surg endosc (2021) 35(3):1156–63. doi: 10.1007/s00464-020-07480-0 32144557

[29] Park YK Yoon HM Kim YW Park JY Ryu KW Lee YJ. Laparoscopy-assisted versus open D2 distal gastrectomy for advanced gastric cancer: Results from a randomized phase II multicenter clinical trial (COACT 1001). Ann surg (2018) 267(4):638–45. doi: 10.1097/SLA.0000000000002168 28187041

[30] Brenkman HJF Gisbertz SS Slaman AE Goense L Ruurda JP van Berge Henegouwen MI. Postoperative outcomes of minimally invasive gastrectomy versus open gastrectomy during the early introduction of minimally invasive gastrectomy in the Netherlands: A population-based cohort study. Ann surg (2017) 266(5):831–8. doi: 10.1097/SLA.0000000000002391 28742708

