# Supplementary material for: Laparoscopic distal gastrectomy demonstrates acceptable outcomes regarding complications compared to open surgery for gastric cancer patients with pylorus outlet obstruction
Source: Front Oncol. 2023 Apr 27;13:1169454. doi: 10.3389/fonc.2023.1169454 (PMC10174232; doi:10.3389/fonc.2023.1169454)
Supplement: Supplementary file 1 [file Table_1.docx]

**Supplementary Table 1** Univariate and multivariate analysis of variance in overall complications between laparoscopic and open surgery.

|  | Variables |  | Complication | | Univariate | Multivariate |
| --- | --- | --- | --- | --- | --- | --- |
|  |  |  | Yes | No | p-Value | p-Value |
| Overall Complication Rates | Surgery |  |  |  |  |  |
|  |  | Laparoscope | 21 | 109 | **0.041^*^** | **0.035^*^** |
|  |  | Open | 29 | 82 |  |  |
|  | T Stage |  |  |  |  |  |
|  |  | T1 | 5 | 12 | 0.264 | / |
|  |  | T2 | 4 | 8 |  | 0.288 |
|  |  | T3 | 13 | 85 |  | 0.392 |
|  |  | T4 | 28 | 86 |  | 0.943 |
|  | N Stage |  |  |  |  |  |
|  |  | N0 | 16 | 45 | 0.199 | / |
|  |  | N1 | 4 | 22 |  | 0.217 |
|  |  | N2 | 5 | 38 |  | 0.130 |
|  |  | N3a | 17 | 45 |  | 0.307 |
|  |  | N3b | 8 | 41 |  | 0.241 |
