# Supplementary material for: Laparoscopic distal gastrectomy demonstrates acceptable outcomes regarding complications compared to open surgery for gastric cancer patients with pylorus outlet obstruction
Source: Front Oncol. 2023 Apr 27;13:1169454. doi: 10.3389/fonc.2023.1169454 (PMC10174232; doi:10.3389/fonc.2023.1169454)
Supplement: Supplementary file 2 [file Table_2.docx]

**Supplementary Table 2** Univariate and multivariate analysis of variance in grade III-V complications between laparoscopic and open surgery.

|  | Variables |  | Complication | | Univariate | Multivariate |
| --- | --- | --- | --- | --- | --- | --- |
|  |  |  | Yes | No | p-Value | p-Value |
| Grade III-V Complication | Surgery |  |  |  |  |  |
|  |  | Laparoscope | 5 | 127 | 0.574 | 0.401 |
|  |  | Open | 2 | 109 |  |  |
|  | T Stage |  |  |  |  |  |
|  |  | T1 | 2 | 15 | **<0.001*** | Reference |
|  |  | T2 | 2 | 10 |  | 0.647 |
|  |  | T3 | 1 | 97 |  | 0.192 |
|  |  | T4 | 0 | 114 |  | 0.195 |
|  | N Stage |  |  |  |  |  |
|  |  | N0 | 4 | 57 | 0.072 | Reference |
|  |  | N1 | 0 | 26 |  | 0.399 |
|  |  | N2 | 0 | 43 |  | 0.992 |
|  |  | N3a | 1 | 61 |  | 0.454 |
|  |  | N3b | 0 | 49 |  | 0.993 |
|  | | | | | | |
