# Supplementary material for: Laparoscopic distal gastrectomy demonstrates acceptable outcomes regarding complications compared to open surgery for gastric cancer patients with pylorus outlet obstruction
Source: Front Oncol. 2023 Apr 27;13:1169454. doi: 10.3389/fonc.2023.1169454 (PMC10174232; doi:10.3389/fonc.2023.1169454)
Supplement: Supplementary file 3 [file Table_3.docx]

**Supplementary Table 3** Univariate and multivariate analysis of variance in anastomosis-related complications.

|  | Variables |  | Complication | | Univariate | Multivariate |
| --- | --- | --- | --- | --- | --- | --- |
|  |  |  | Yes | No | p-Value | p-Value |
| Anastomosis-related Complication | Surgery |  |  |  |  |  |
|  |  | Laparoscope | 4 | 126 | 0.587 | 0.178 |
|  |  | Open | 3 | 108 |  |  |
|  | T Stage |  |  |  |  |  |
|  |  | T1 | 1 | 16 | 0.337 | Reference |
|  |  | T2 | 1 | 11 |  | 0.556 |
|  |  | T3 | 1 | 97 |  | 0.996 |
|  |  | T4 | 4 | 110 |  | 0.849 |
|  | N Stage |  |  |  |  |  |
|  |  | N0 | 4 | 57 | 0.150 | Reference |
|  |  | N1 | 1 | 25 |  | 0.750 |
|  |  | N2 | 2 | 41 |  | 0.439 |
|  |  | N3a | 0 | 62 |  | 0.713 |
|  |  | N3b | 0 | 49 |  | 0.997 |
